# Exploring the Association Between Vitamin D and IL-10 in Allergic Parthenium Contact Dermatitis Patients

**DOI:** 10.7759/cureus.85515

**Published:** 2025-06-07

**Authors:** Alphienes Stanley Xavier, Sandhiya Selvarajan, Saibal Das, Sadishkumar Kamalanathan

**Affiliations:** 1 Pharmacology, All India Institute of Medical Sciences, Madurai, Madurai, IND; 2 Clinical Pharmacology, Jawaharlal Institute of Postgraduate Medical Education and Research, Puducherry, IND; 3 Public Health, Indian Council of Medical Research, Kolkata, IND; 4 Endocrinology, Jawaharlal Institute of Postgraduate Medical Education and Research, Puducherry, IND

**Keywords:** allergy, anti-inflammatory cytokine, eczema, parthenium dermatitis, sunshine vitamin

## Abstract

Introduction

The imbalance between pro- and anti-inflammatory mediators was suggested to be a contributory factor to the manifestations of allergic parthenium dermatitis. Inadequate circulating vitamin D and IL-10 levels can significantly influence the course of this allergic dermatitis.

Objective

The objective was to study the association between circulating IL-10 levels and vitamin D status in patients with parthenium dermatitis.

Materials and methods

Patients attending the dermatitis clinic were screened for eligibility, and 88 individuals were recruited. A total of 101 unrelated healthy volunteers were included as controls. Circulating IL-10 cytokine and vitamin D levels were determined in both groups and compared.

Results

A higher prevalence of vitamin D deficiency (79.5% vs. 59.4%, P = 0.000315) and lower IL-10 levels (6.84 vs. 9.04 pg/ml, P < 0.0001) were observed in the patient group compared to healthy controls. The vitamin D-deficient patients were also found to have significantly lower IL-10 levels. A significant positive correlation between vitamin D and IL-10 levels was observed among individuals with allergic dermatitis. Lower vitamin D and IL-10 levels were associated with higher Dermatology Life Quality Index (DLQI) scores.

Conclusion

Lower circulating vitamin D and IL-10 levels, observed in patients with parthenium dermatitis, significantly impacted their quality of life. Assessing plasma IL-10 levels could be a potential biomarker for evaluating disease severity and treatment efficacy. Correcting vitamin D deficiency may improve IL-10 levels and enhance treatment outcomes.

## Introduction

*Parthenium hysterophorus* is a common weed plant in India that belongs to the *Compositae* family. The weed plant, which is also known as "congress weed," can cause a chronic relapsing allergic skin condition commonly called parthenium dermatitis. It is a prevalent condition among the Indian population, and most cases are found among farmers who are exposed to the weed's allergens in their occupational environment [1]. In India, Parthenium is the most common allergen that causes contact dermatitis [2]. The disease runs a chronic course with frequent relapses on exposure to allergens and significantly affects the quality of life of individuals affected. The significant allergens involved are sesquiterpene lactones [3,4]. The disease can be presented as a classical airborne pattern, a chronic actinic pattern, or a mixed pattern [5]. The manifestations were explained as due to a combined type IV and type I hypersensitivity reaction. Researchers have observed a significant imbalance between pro-inflammatory and anti-inflammatory cytokines, which could implicate the pathogenesis of this condition [6,7].

IL-10 is an important anti-inflammatory cytokine that plays a crucial role in the smooth resolution of inflammatory responses and the prevention of tissue damage by regulating multiple immune cells [8]. Circulating levels of IL-10 were found to be significantly lower in parthenium dermatitis patients, and the genetic mutations in the IL-10 gene associated with lower cytokine production were found to be of higher frequency among them compared to controls [9]. IL-10 deficiency was found to be related to exaggerated Th1 response, as observed in inflammatory conditions and autoimmune diseases. In contrast, the overexpression of IL-10 would lead to a skewed Th2 response, as observed with systemic lupus erythematosus and bronchial asthma [10].

The role of vitamin D, the fat-soluble vitamin, extends beyond calcium homeostasis and bone mineralization. The level of its circulating metabolite indicates the adequacy of the vitamin D storage pool in the body, 25-OH vitamin D. In a systematic review conducted by Hilger et al., the prevalence of vitamin D deficiency was reported to be 37.3% based on studies worldwide [11]. The deficiency is common in India, with a frequency of 70-90% in all age groups, regardless of gender [12]. The adverse consequence of deficiency is not only associated with bone health but also with immunological disorders, infectious diseases, cardiovascular conditions, diabetes, and cancer [13].

The optimal circulating level for vitamin D has been defined, considering its skeletal benefits. The cutoff levels suggested for vitamin D status classification may not be appropriate for people from all ethnicities or the vitamin's extra-skeletal benefits. Recently, vitamin D has been viewed as a negative acute-phase reactant, and reduced levels may be a sequela rather than a causative factor for associated conditions [14]. In published preclinical and clinical studies, vitamin D has been shown to induce IL-10 expression and its production by immune cells [15]. These studies revealed a positive correlation between serum vitamin D levels and IL-10 levels.

With this background, this research was conducted to investigate the association between circulating IL-10 levels and vitamin D status in patients with allergic contact dermatitis following exposure to parthenium.

## Materials and methods

Study setting and population

The research was carried out by the Department of Clinical Pharmacology and the Department of Dermatology. The study procedures followed were in accordance with the ethical standards of the Institutional Ethics Committee (IEC) for human studies, Jawaharlal Institute of Postgraduate Medical Education and Research (JIPMER), and with the principles outlined in the Declaration of Helsinki. The study protocol was approved by the IEC for human studies. Patients attending the Dermatology Outpatient Department (OPD) were screened for study eligibility. Clinically diagnosed patients with characteristic lesions over the exposed body areas suggestive of allergic parthenium dermatitis, treatment-naïve, of any gender, aged 30 to 60 years, were included in the study. Patients already consuming vitamin D supplements and individuals with renal or hepatic dysfunction were excluded. After explaining the study details, written informed consent was obtained from the willing individuals. Data on healthy controls used in this study for comparison were obtained from our previously published work [16].

Disease severity and quality of life assessment

The Eczema Area Severity Index (EASI) was used to assess the severity of dermatitis. This scale provides a composite score considering the extent of the body surface involved and the severity of the lesions. Based on the scores of 1.1-7, 7.1-21, 21.1-50, and 50.1-72, cases were classified as mild, moderate, severe, and very severe, respectively [17]. The Dermatology Life Quality Index (DLQI) was used to assess the impact on quality of life, comprising 10 questions with a maximum score of 3 for each question [18].

Vitamin D and IL-10 measurement

A total blood volume of 6 ml was collected from parthenium dermatitis patients and healthy controls. Measurement of serum vitamin D levels was done using the Siemens-ADVIA Centaur XP Immunoassay system (Erlangen, Germany) with a chemiluminescence assay. The assay procedure was standardized using known references in accordance with the vitamin D standardization program for each run. The assay has a detection limit from 4.2 ng/mL to 150 ng/mL.

Circulating IL-10 cytokine levels were measured in plasma using the ELISA (enzyme-linked immunosorbent assay) method, with absorbance at 450 nm determined following the manufacturer's guidelines (RayBiotech, Peachtree Corners, GA, USA). A standard curve of concentration versus absorbance was plotted to calculate IL-10 concentrations in the plasma samples. The assay was performed in duplicate, and the average absorbance values were used for analysis.

Statistical analysis

Medians (interquartile range) or numbers (percentages) were used to express descriptive statistics. Since most of the parameters did not pass normality by the Kolmogorov-Smirnov test, non-parametric statistical tests were used for analysis. The Mann-Whitney test was used to compare between different groups or categories. Spearman correlation was used to study the association between disease severity indices and vitamin D or IL-10 levels. A P-value below 0.05 was considered to demonstrate statistical significance. The data were analyzed using IBM SPSS Statistics for Windows, Version 27 (Released 2020; IBM Corp., Armonk, New York).

## Results

Patients attending the dermatitis clinic of the Dermatology OPD were screened for eligibility as per the predefined eligibility criteria. A total of 145 patients with allergic contact dermatitis to parthenium were screened, and 88 patients were included after obtaining written informed consent. A total of 101 apparently healthy volunteers were included as controls.

Table 1 presents the demographic characteristics, IL-10, and vitamin D levels of the participants. The median age of dermatitis patients was 56 years, and 89.8% were males; 79.5% of them were found to be vitamin D deficient compared to 59.4% among the healthy controls. The median IL-10 levels in the patient group were significantly lower than those in the control group (6.84 vs. 9.04 pg/ml).

**Table 1:** Demographic characteristics of the study participants * P < 0.0001, Mann-Whitney test; comparison of median IL-10 levels between the patient group and healthy controls. ^†^ P = 0.000315, Chi-square test; comparison of proportion. Values are expressed as medians (IQR). Gender is expressed as male, N (%). BMI: body mass index; IL-10: interleukin 10; IQR: interquartile range; SD: standard deviation

Characteristic	Patient Population (N = 88)	Healthy Controls (N = 101)	
			Age (years)	56 (47.25, 59)	32 (28, 43)	
Gender (male, %)	79 (89.8%)	44 (43.6%)	
Weight (kg)	55 (48.25, 59)	64 (55, 71.5)	
BMI (kg/m^2^)	21.40 (19.32, 22.86)	24.49 (21.73, 26.74)	
Sun exposure (in hours)	3 (1, 3)	2 (0.63, 4.0)	
IL-10 (pg/ml)	6.84 (4.12, 11.06)*	9.04 (7.75, 11.34)*	
Vitamin D (ng/ml), median (IQR)	18.02 (14.9, 19.70)	18.21 (11.3, 23.08)	
Mean (SD)	20.50 (9.91)	17.88 (7.18)	
Vitamin D deficiency, n (%)	70 (79.5%)^†^	60 (59.4%)^†^	

The disease severity was assessed using the EASI scale. All patients were classified into two distinct categories: "moderate" or "severe." A total of 77 out of 88 patients had EASI scores between 7.1 and 20, thereby classifying them as having "moderate" severity (Table 2). In 78.4% of the study participants, the disease greatly affected their quality of life, with their DLQI scores falling between 11 and 20. There was no significant difference in either the IL-10 or vitamin D levels between the moderate and severe dermatitis groups.

**Table 2:** Disease severity and quality of life index among the patient population Values are expressed as medians (IQR). P > 0.05, Mann-Whitney test; comparison of vitamin D and IL-10 levels between moderate and severe (large) categories. EASI: Eczema Area Severity Index; DLQI: Dermatology Life Quality Index

Disease Index	n (%) (N = 88)	Vitamin D	IL-10
EASI			
7.1–21 (moderate)	77 (87.5)	18.17 (15.25, 19.76)	6.69 (4.09, 11.06)
21.1–50 (severe)	11 (12.5)	16.4 (14.52, 17.60)	7.0 (6.0, 10.44)
DLQI			
6–10 (moderate effect)	19 (21.6)	18.07 (13.52, 38.75)	7.94 (6.69, 12.94)
11–20 (large effect)	69 (78.4)	18.00 (15.55, 19.57)	6.69 (4.00, 10.75)

**Figure 1:**
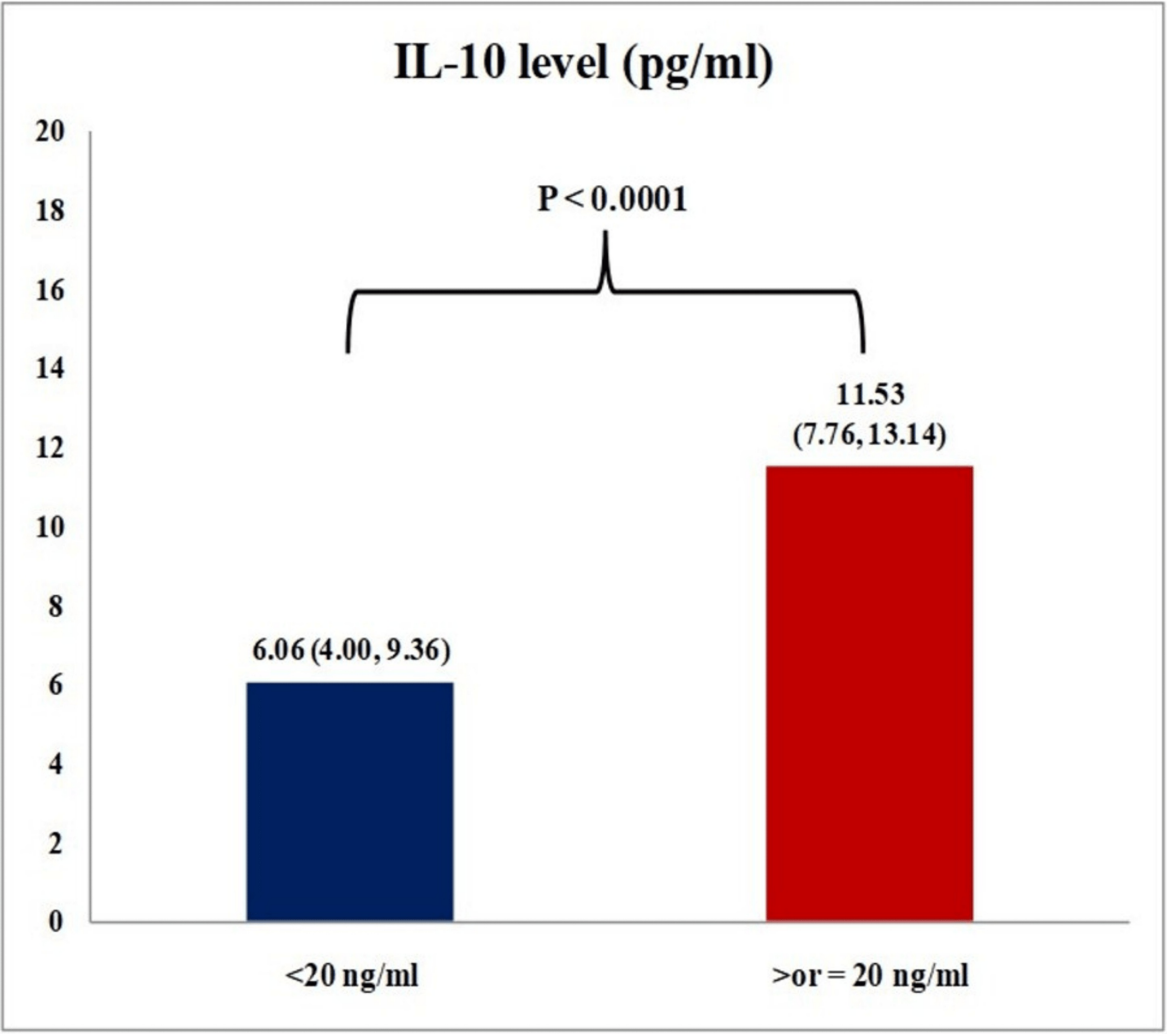
Vitamin D status and IL-10 level in patients with parthenium dermatitis (N = 88) IL-10 levels are expressed as medians (IQR); P < 0.0001 (Mann-Whitney test).

The median plasma IL-10 levels of vitamin D deficient (<20 ng/ml) and non-deficient (≥20 ng/ml) are presented in Figure 1. The correlation between disease severity, quality of life scales, and levels of vitamin D and IL-10 was analyzed using a non-parametric Spearman correlation test (Table 3). A significant positive correlation between vitamin D and IL-10 levels was observed among individuals with allergic dermatitis. Lower vitamin D and IL-10 levels were associated with higher DLQI scores, signifying their impact on quality of life.

**Table 3:** Correlating vitamin D and IL-10 levels with disease severity among parthenium dermatitis patients A non-parametric Spearman correlation was conducted. EASI: Eczema Area Severity Index; DLQI: Dermatology Life Quality Index

Parameter	IL-10	Vitamin D
EASI	0.006 (P = 0.956)	-0.061 (P = 0.571)
DLQI	-0.232 (P = 0.029)	-0.239 (P = 0.025)
Vitamin D	0.405 (P < 0.0001)	-

## Discussion

Of the 88 patients recruited for the study, 54 were agricultural workers (61.4%). Exposure to allergens in the workplace increased the risk of developing allergic dermatitis. Parthenium allergic dermatitis was found to be common in the middle and older age groups. The mean age of our study's patient population was 56 years, with a range of 27 to 60 years. Consistent with previous reports, male preponderance was observed, with 89.8% of the patient population being males [19].

Significantly lower levels of IL-10 were noted among the disease group compared to controls (6.84 vs. 9.04 pg/ml). The result was similar to the observations made by Akhtar et al. (2010) [7] and Khatri et al. (2011) [9]. Akhtar et al. demonstrated that the Th2 cytokine IL-10 was significantly less in parthenium-induced contact dermatitis patients (4.15 vs. 7.94 pg/ml in controls) [7]. In a study by Khatri et al. (2011), the mean IL-10 level was 4.15 pg/ml in dermatitis patients compared to the control group (8.23 pg/ml) [9]. As a vital anti-inflammatory cytokine, insufficient levels of IL-10 can shift the balance toward pro-inflammation, potentially worsening the inflammatory consequences of disease conditions.

As per Endocrine Society clinical practice guidelines, a serum 25(OH) vitamin D level less than 20 ng/ml (50 nmol/l) is defined as vitamin D deficiency, and levels between 21 and 29 ng/ml are defined as vitamin D insufficiency [20]. In our study, we observed that 59.4% of the individuals among healthy controls and 79.5% of parthenium patients had vitamin D deficiency. The patients were advised to avoid sunlight exposure as a preventive measure to prevent worsening, and the diseased skin had a reduced ability to synthesize vitamin D. These factors may contribute to the high prevalence of vitamin D deficiency in the patient group. However, no statistically significant difference was identified between the average vitamin levels of the patients and those of the healthy controls. The mean vitamin D levels among the healthy Indian population were reported to be between 3.19 and 52.9 ng/ml, with a frequency of 70-100% [21]. A meta-analysis conducted at our center reported that vitamin D levels among the Indian population from different geographic zones varied from 12.28 to 17.45 ng/ml, with an overall value of 14.16 ng/ml among the healthy population [22].

The control group was not age- and gender-matched with the patient group. Vitamin D levels tend to be higher among males compared to females [22]. Since the patient group consists predominantly of males, this could be a reason for the insignificant difference in vitamin D levels between the patient and control groups.

The median circulating IL-10 levels were compared between individuals with vitamin deficiency and non-deficient patients. Significantly lower IL-10 levels were seen in the deficient group (6.06 pg/ml). The relationship between the sunshine vitamin and the anti-inflammatory cytokine has been well documented in in vitro, preclinical, and clinical studies. A study investigating the potential mechanism of vitamin D analogs in psoriasis revealed that calcitriol and calcipotriol induce the expression of the IL-10 receptor, and the receptor gene exhibited responsive elements in its promoter region [23]. In an in vitro study conducted by Bakdash et al. [24], vitamin D analogs inhibited the maturation of dendritic cells, decreased the production of TNF-α, and increased IL-10 production. Moreover, dendritic cells primed with calcitriol promoted the development of Treg cells expressing IL-10 [24]. Significant improvement in IL-10 levels was noted after vitamin D supplementation in inflammatory conditions with multiple sclerosis and congestive heart failure, and this improvement also helped to bring immune balance in controlling inflammation [25-27]. In a clinical trial conducted at our center, vitamin D supplementation in deficient individuals with allergic dermatitis displayed a trend toward improving IL-10 levels and quality of life [28].

Several malignancies, including melanoma, squamous cell carcinoma, basal cell carcinoma, and lymphomas, have been shown to overexpress IL-10. Elevated IL-10 levels have been linked to a poor prognosis, specifically in malignant lymphomas. The role of IL-10 in allergic contact dermatitis was realized through findings such as the inhibition of the effector phase of the reaction by IL-10 administration and the ability of therapeutic ultraviolet (UV) therapy to increase the production and clearance of UV-induced dermatitis mediated by IL-10. In allergic disorders such as atopic dermatitis and asthma, overexpression of IL-10 has been considered a counter-regulatory response to limit inflammation. The beneficial effects of steroids and anti-leukotrienes in asthma could be due to their ability to increase serum IL-10 levels. Based on these findings, IL-10 was evaluated as a therapeutic target, and recombinant IL-10 therapy has been attempted in rheumatoid arthritis, Crohn's disease, and HIV infection; however, the results have been unsatisfactory. IL-10 therapies in psoriasis vulgaris and associated arthritis were found to be beneficial with good to moderate responses [29].

Serum vitamin D and plasma IL-10 levels did not significantly differ between moderate and severe dermatitis patients. EASI was also not significantly correlated with vitamin D and IL-10 levels. Nevertheless, a significant negative correlation was noted between the vitamin D and IL-10 levels and DLQI scores. Lower vitamin D and IL-10 levels may have a greater impact on the quality of life among individuals with allergic dermatitis. Larger sample size studies could facilitate the study of the correlation between IL-10 or vitamin D levels and the severity of the disease. IL-10 could also be evaluated as a potential biomarker for assessing clinical improvement after treatment initiation.

Limitations

We considered the following as limitations to our study: we included only patients with clinically diagnosed parthenium dermatitis. Patch test positivity was not considered a confirmatory test for subject inclusion; we did not perform a sample size calculation. All patients who attended the outpatient department (OPD) during the study period were screened for eligibility. The control group was not age- and gender-matched with the patient group. We have also not studied potential confounding factors, such as sunlight exposure, nutritional status, and occupation, among the study population, which may influence vitamin D levels.

## Conclusions

Compared to healthy controls, patients with parthenium dermatitis had significantly lower levels of IL-10. Among them, vitamin D-deficient patients had significantly lower circulating IL-10 levels. Circulating vitamin D and IL-10 levels were negatively correlated with the DLQI. Hence, assessing plasma IL-10 levels could be a potential biomarker for evaluating disease severity and treatment efficacy. Correcting vitamin D deficiency may help enhance IL-10 levels, leading to improved treatment outcomes.
